# Microbial Primer: Lipopolysaccharide – a remarkable component of the Gram-negative bacterial surface

**DOI:** 10.1099/mic.0.001439

**Published:** 2024-03-07

**Authors:** Leah M. VanOtterloo, M. Stephen Trent

**Affiliations:** 1 Department of Microbiology, College of Arts and Sciences, University of Georgia, Athens, Georgia, USA; 2 Department of Infectious Diseases, College of Veterinary Medicine, University of Georgia, Athens, Georgia, USA

**Keywords:** LPS, LOS, lipopolysaccharide, lipooligosaccharide, lipid A, outer membrane

## Abstract

Lipopolysaccharide (LPS) is a fundamental tripartite glycolipid found on the surface of nearly all Gram-negative bacteria. It acts as a protective shield for the bacterial cell and is a potent agonist of the innate immune system. This primer serves to introduce the basic properties of LPS, its function in bacterial physiology and pathogenicity, and its use as a therapeutic target.

## History and discovery of LPS

The discovery of LPS is largely attributed to the physician and microbiologist Richard Pfeiffer. While researching the pathogenesis of *Vibrio cholerae* during an outbreak of cholera in the late 19th century, he observed that *Vibrio* cells that had been heat-killed were just as immunogenic as living cells, resulting in fever, shock, and even death. Notably, heat-killing of bacterial cells induces lysis and therefore loss of cytoplasm, but maintains other major physiological components such as the cellular membranes and cell wall. Pfeiffer’s results were quite groundbreaking at the time, namely because they disagreed with the famed postulates of his own mentor, Robert Koch, which dictated in part that a living microbe must be recovered from a host in order to establish the microbe as the causative agent of a disease. Of course, as the microbe had been heat-killed, it could not be recovered from the diseased host. Since the heat-killed bacteria had lost its cytoplasm and therefore could not reasonably generate a secreted toxin, Pfeiffer speculated that this novel toxin must be physically linked to the bacterial cell itself and thus named it ‘endotoxin’.

Numerous researchers continued studying this so-called endotoxin in the years that followed. With a better understanding of the molecule itself, the concept of endotoxin eventually transformed into what we now refer to as lipopolysaccharide (LPS), a much more apt and straightforward description of this biologically important molecule. Decades of multidisciplinary research have made huge strides in broadening our understanding of LPS, leading to the elucidation of its overall structure, its role in bacterial physiology and its impact on human health and disease.

## LPS structure

**Fig. 1.**
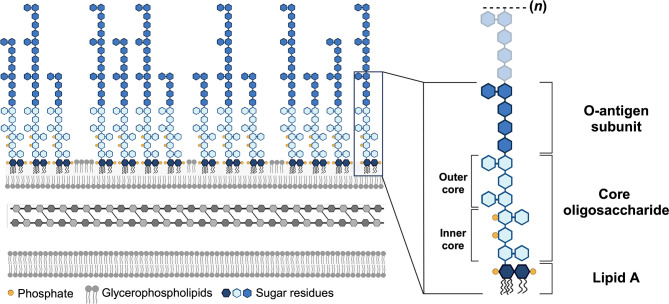
Schematic representation of the Gram-negative cell envelope and LPS structure. The Gram-negative cell envelope consists of three primary layers (left). The inner membrane is a symmetric glycerophospholipid bilayer that is separated from the outer membrane by a thin layer of peptidoglycan-based cell wall located in the periplasm. The outer membrane is an asymmetric bilayer with glycerophospholipids in the inner leaflet and primarily LPS in the outer leaflet. The single magnified LPS molecule (right) is made up of lipid A, a non-repeating core oligosaccharide, and the O-antigen domain comprised of a repeating polymer of variable lengths, represented by (***n***). Lipid A is a disaccharide of glucosamine that is both fatty acylated and phosphorylated. The hexa-acylated *bis*-phosphorylated lipid A structure of *E. coli* is shown. Created in part using BioRender.com.

LPS is composed of three domains that are each unique to a bacterial species and, in many cases, are also unique to a bacterial strain. These components are a glycolipid termed lipid A, a core oligosaccharide, and an O-antigen polymer (Fig. 1).

The lipid A glycolipid – the most conserved portion of LPS – anchors the entire LPS molecule onto the surface (discussed below) of Gram-negative bacteria and contributes greatly to the negative charge of the molecule. The conserved structure of lipid A is a *bis*-phosphorylated disaccharide of glucosamine decorated with multiple fatty acids that are fully saturated (Fig. 1). Typically, the number of chains ranges from 4 to 7 with lengths of 10 to 16 carbons. In the model organism *Escherichia coli*, for example, the lipid A molecule contains six acyl chains made up of 12 or 14 carbons. The number of acyl chains in a lipid A molecule, as well as the length of each chain, is highly conserved within the same species but more diverse among different species.

The core oligosaccharide is a short series of 8–12 sugar residues that extend from the lipid A region. This component can be divided into two sections – the somewhat uniform inner core and the highly variable outer core (Fig. 1), which differs even among strains within a bacterial species. In most bacteria, the inner core begins with at least one 3-deoxy-d-*manno*-octulosonic acid (Kdo) residue linked to the glucosamine of lipid A. This is typically followed by several heptoses as well as other branching sugars to complete the inner core. In many cases, these heptose residues are phosphorylated, providing additional negative charges to the LPS molecule. Negatively charged sugar residues such as Kdo or hexuronic acids contribute similar anionic effects to LPS. While this is the most common configuration of the inner core, the core oligosaccharide is quite diverse and there are numerous variations on this inner core archetype. Several examples of heptose-deficient and/or phosphate-deficient core oligosaccharides exist, particularly in non-enteric bacteria, resulting in a somewhat arbitrary designation of the inner core region in those organisms. In fact, the only component of the core oligosaccharide conserved among all known bacterial core oligosaccharides is the presence of at least one lipid A-linked Kdo residue or, in very few cases, the Kdo analogue d-*glycero*-d-*talo*-oct-2-ulosonic acid (Ko).

The terminal domain of LPS is the O-antigen (Fig. 1), a lengthy polysaccharide made up of repeating subunits. These subunits are mainly comprised of monosaccharides but also commonly include non-carbohydrate residues such as phosphate groups, acetyl groups and even amino acids. Importantly, the O-antigen is highly structurally diverse both within and among bacterial species – for example, over 180 unique structures have been discovered thus far in *E. coli* alone. Isolates within the same strain can even display varying lengths of O-antigen polymers, indicating differing numbers of repeat units. This extensive structural heterogeneity helps facilitate the precise classification of bacteria within the same species based on their unique O-antigen composition, making the O-antigen a useful tool in serotyping schemes, taxonomy, epidemiology and vaccine development. The possession of O-antigen is predominant in LPS, but the complete structure of all three components – lipid A, core oligosaccharide and O-antigen – technically indicates a particular subtype of LPS called ‘smooth LPS’. This designation refers to the early observation that organisms with this type of LPS form smooth colonies when grown on agar plates. LPS lacking the O-antigen component is referred to as ‘rough LPS’. As a point of distinction, organisms that lack the innate ability to synthesize the O-antigen component possess lipooligosaccharide (LOS) instead of the canonical LPS.

## LPS function

The hallmark of a Gram-negative bacterial cell is the presence of a second cellular membrane called the ‘outer membrane’. Instead of a symmetric lipid bilayer, the outermost environment-facing leaflet of the outer membrane is rich in LPS, while the inner leaflet comprises glycerophospholipids (Fig. 1). Thus, unlike the glycerophospholipid bilayer of the inner membrane, the outer membrane is asymmetric in nature. This unique structure affords an additional layer of protection that prevents penetration of not only polar solutes, but hydrophobic compounds as well. Indeed, an asymmetric outer membrane is widely considered to be essential for survival of Gram-negative bacteria due to the innate protection from toxic compounds afforded by an outer leaflet made primarily of LPS.

Much of the protective quality of LPS is provided by its lipid A moiety. A principal feature of this glycolipid anchor is the presence of numerous lengthy saturated fatty acid chains. These saturated fatty acid chains interact with one another closely, leading to a densely packed layer of LPS. This results in an outer membrane with increased hydrophobicity and decreased fluidity in comparison to the inner membrane. However, like glycerophospholipids, lipid A acylation can be altered in a process known as homeoviscous adaptation in response to shifts in temperature. This leads to incorporation of acyl chains with unsaturations or fatty acids of reduced length, helping to maintain the optimal outer membrane barrier.

Another important aspect of membrane asymmetry is the negative charge afforded by the LPS molecule. As discussed in the previous section, there are multiple opportunities for addition of negative charges via phosphorylation of the lipid A moiety, as well as phosphorylation or addition of negatively charged sugar residues to the core oligosaccharide and, to some extent, the O-antigen. These negative charges importantly attract divalent cations from the surrounding environment to form stabilizing cross-bridges between adjacent LPS molecules. When these cross-bridges form between LPS molecules at the surface of the cell, this stabilization is multiplied and applied to the entire outer membrane, yielding a much more robust and fortified barrier that stabilizes the cell and prevents entry of harmful compounds.

## LPS modifications and antibiotic resistance

The negative charge of LPS is undoubtedly beneficial to the bacterial cell, which makes this molecule a clear candidate for exploitation as an impactful antibiotic target. The most notable and straightforward group of antibiotics to target LPS directly are members of a group called cationic antimicrobial peptides (CAMPs). CAMPs are small, amphipathic compounds that are ubiquitous in nature and part of the innate immune system of higher organisms. These peptides bind to the negatively charged components of the bacterial surface, including LPS, and disrupt cell envelope structure, promoting cell lysis. Examples of CAMP antibiotics include polymyxin B and polymyxin E (colistin). Despite the designation of polymyxin B and E as ‘last-resort’ antibiotics due to their high occurrence of nephrotoxicity, they continue to grow in popularity as multidrug antibiotic resistance becomes more urgent.

As is often the case, Gram-negative bacteria have amassed a toolbox of methods to combat these antibiotics that mostly revolve around reducing the negative charge that attracts CAMPs. Although this is a very direct strategy, these bacteria use numerous unique methods of achieving it. The simplest technique is the phosphatase-catalysed removal of phosphate groups on lipid A, which directly reduces the negative charge of the glycolipid. Numerous positively charged groups, including but not limited to phosphoethanolamine, aminoarabinose and glucosamine, can be transferred onto the lipid A molecule by specialized transferases that help to neutralize the negative charge of the outer membrane. Certain organisms can also modify the lipid A with a positively charged amino acid that imparts a similar neutralizing effect. While the majority of discovered LPS alterations occur on the lipid A domain, these changes are not limited to the glycolipid and covalent modification of inner core oligosaccharide is also common.

The most dramatic method of antibiotic resistance via LPS modification is the removal of it – or rather, the prevention of its synthesis via mutations in early lipid A synthesis. Loss of the lipid A anchor prevents addition of core oligosaccharide and O-antigen moieties and results in a total lack of the LPS molecule. A small number of bacteria can survive in the absence of LPS via laboratory-directed mutations, but this phenomenon has only been seen as an antibiotic resistance mechanism in the organism *Acinetobacter baumannii*. Of note, *A. baumannii* lacks O-antigen synthesis capabilities and therefore possesses LOS instead of LPS (see ‘LPS structure’). Although a significant increase in resistance to LPS/LOS-targeting polymyxin antibiotics can be achieved through loss of LOS, it comes at a steep cost to the bacterial cell itself. LOS-deficient *A. baumannii* exhibits slowed growth, decreased virulence and hypersensitivity to antibiotics typically ineffective against Gram-negative organisms. This unique phenotype provides insight into an exciting field of research studying the relationship between LPS/LOS in the outer membrane and antibiotic resistance.

## Role of LPS in health and disease

LPS not only plays a pivotal role for the Gram-negative bacterial cell itself, but it also represents an important intersection between the bacterium and an infected host. When discussing the role of LPS in health and disease, the molecule is commonly referred to as ‘endotoxin’. As explained in the first section, Pfeiffer frequently observed dire inflammatory reactions in subjects exposed to high amounts of endotoxin. We now know that the pyrogenicity originating from endotoxin is a downstream result of the interaction between the lipid A domain of LPS and the human Toll-like receptor 4/myeloid differentiation factor 2 (TLR4/MD-2). Many cell types of the innate immune system, such as monocytes, lymphocytes and endothelial cells, possess TLR4/MD-2. These receptors are able to detect even picomolar levels of endotoxin, making them a highly sensitive warden of the immune system. When large quantities of LPS are released into the host environment, perhaps as a result of an infection (i.e. more bacteria will shed more LPS), activation of TLR4/MD-2 results in a dramatic immune response, yielding dangerously high fevers, system-wide inflammation, septic shock and even death.

TLR4/MD-2 is not only highly sensitive to the quantity of LPS, but also to the composition of LPS. This provides an opportunity for both commensal and pathogenic bacteria to evade the host immune response via LPS modifications. For example, removal of phosphate groups from the lipid A moiety reduces its attraction to TLR4/MD-2, thus reducing detection sensitivity. Bacteria are similarly able to reduce the number of acyl chains of lipid A, which decreases the hydrophobicity of the molecule and yields weaker interactions with the hydrophobic pocket of TLR4/MD-2. Both strategies in addition to other LPS modifications not discussed here can alter the immune response and impact bacterial survival in the host.

Researchers have smartly used this high specificity of TLR4/MD-2 and the resulting immune response to our advantage in vaccine development. A key aspect of a successful vaccine is the adjuvant – a compound that helps prime the immune system to elicit a robust response to the vaccine antigen of choice without overwhelming the host. One adjuvant system in clinical use is a heterogenous mixture of chemically detoxified lipid A. By removing key phosphate groups and acyl chains, these detoxified lipids allow for a significantly dampened yet effective immune response compared to highly toxic native LPS. Components of the LPS structure also serve as targeted antigens. These vaccines have primarily focused on the O-antigen due to its inherently non-toxic yet immunostimulatory properties. A key hurdle in using the O-antigen domain, however, lies in its extensive diversity across bacterial strains. This warrants the need for more complex ‘multivalent’ glycoconjugate vaccines that include multiple O-antigen serotypes, many of which show promise after successful preclinical and clinical trials.

## Conclusion

LPS is a complex glycolipid that provides protective asymmetry to the outer membrane of Gram-negative bacteria. Despite the advent of effective LPS-targeting antibiotics (e.g. polymyxins) over the past century, the arsenal of methods that bacteria have developed to alter their LPS structure and develop resistance to these drugs demands innovative techniques to combat antibiotic resistance. Further studies of LPS structure, function and impact on health and disease will be helpful in understanding bacterial pathogenesis and facilitating the rapid development of new therapeutics.

## Further reading

1. Whitfield C, Trent MS. Biosynthesis and export of bacterial lipopolysaccharides. *Annu Rev Biochem* 2014;83:99–128.

2. Bertani B, Ruiz N. Function and biogenesis of lipopolysaccharides. *EcoSal Plus* 2018;8:10.1128/ecosalplus.ESP-0001–2018.

3. Raetz CRH, Whitfield C. Lipopolysaccharide endotoxins. *Annu Rev Biochem* 2002;71:635–700. 10.1146/annurev.biochem.71.110601.135414.

4. Whitfield C, Williams DM, Kelly SD. Lipopolysaccharide O-antigens-bacterial glycans made to measure. *J Biol Chem* 2020;295:10593–10609.

5. Needham BD, Trent MS. Fortifying the barrier: the impact of lipid A remodelling on bacterial pathogenesis. *Nat Rev Microbiol* 2013;11:467–481.

6. Simpson BW, Trent MS. Pushing the envelope: LPS modifications and their consequences. *Nat Rev Microbiol* 2019;17:403–416.

7. Moffatt JH, Harper M, Boyce JD. Mechanisms of polymyxin resistance. In: Li J, Nation RL and Kaye KS (eds). *Polymyxin Antibiotics: From Laboratory Bench to Bedside*. Cham: Springer International Publishing; 2019. pp. 55–71.

8. Powers MJ, Trent MS. Expanding the paradigm for the outer membrane: acinetobacter baumannii in the absence of endotoxin. *Mol Microbiol* 2018;107:47–56. https://doi.org/10.1111/mmi.13872.

9. Munford RS. Severe sepsis and septic shock: the role of gram-negative bacteremia. *Annu Rev Pathol* 2006;1:467–496.

10. Cross AS. Hit’em where it hurts: gram-negative bacterial lipopolysaccharide as a vaccine target. *Microbiol Mol Biol Rev* 2023;87:e00045-22.

## Abbreviations

CAMP: cationic antimicrobial peptide
Kdo: 3-deoxy-D-*manno*-octulosonic acid
Ko: D-*glycero*-D-*talo*-oct-2-ulosonic acid
LOS: lipooligosaccharide
LPS: lipopolysaccharide
TLR4/MD-2: toll-like receptor 4/myeloid differentiation factor 2

